# Single tooth torque correction in the lower frontal area by a completely customized lingual appliance

**DOI:** 10.1186/s13005-017-0152-2

**Published:** 2017-10-10

**Authors:** Collin Jacobs, Milena Katzorke, Dirk Wiechmann, Heiner Wehrbein, Rainer Schwestka-Polly

**Affiliations:** 1 Department of Orthodontics, University Mainz, Augustusplatz 2, 55131 Mainz, Germany; 2 Private Practice, Bad Essen, Essen, Germany; 3 Department of Orthodontics, Hannover Medical School, Hannover, Germany

**Keywords:** Torque correction, Lingual appliance, Periodontal recession, Root resorption

## Abstract

**Background:**

Aim of this study was to analyze the efficacy and precision of the completely customized lingual appliance (CCLA) regarding the single tooth torque correction. The study also examined external apical root resorptions as possible side effects of torque correction and the changings of the periodontal situation.

**Methods:**

A case series of three patients were included. The patients showed a single tooth torque problem with a gingival recession and were treated with the CCLA. Plaster casts before and after treatment and plaster casts of the set up were scanned and superimposed. Deviations between the two plaster casts were analyzed at different points of interest. Changes of the gingival recession were compared before and after treatment. Relative root resorptions were measured by the orthopantomograms. Treatment times were assessed by the records of the patients. Results were presented descriptively.

**Results:**

The mean change of the most apical part of the root reached by the orthodontic treatment was 1.8 ± 0.3 mm. The largest deviation between set up and final model was measured on the occlusal surface of the tooth 36 with 0.8 mm. Most measurement points showed a deviation of 0.5 mm or less. The depths of the gingival recession showed a significant reduction of 4.7 mm. The widths of the gingival recession were reduced by 1.1 mm. The average relative root resorption of the corrected teeth was 2.7 ± 1.5%. The average treatment time was 13.8 ± 4.5 months.

**Conclusions:**

This is the first study showing that the CCLA with its high precision is very effective in correcting single tooth torque problems. Orthodontic torque correction resulted in a significant reduction of gingival recessions and caused only negligible root resorptions.

## Background

**Fig. 1:**
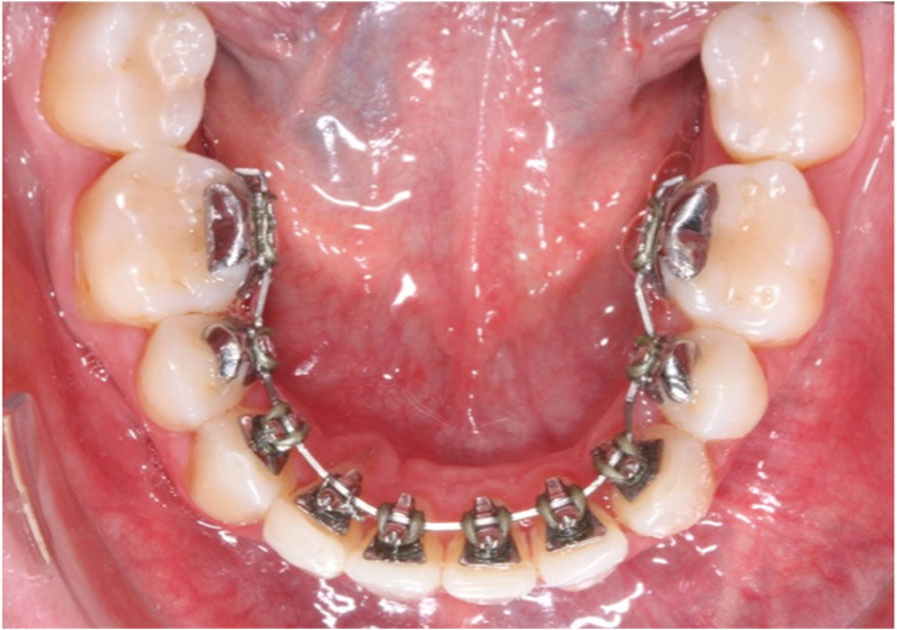
A completely customized lingual appliance (WIN, DW Lingual Systems, Bad Essen, Germany) bonded to the lingual surfaces of the teeth in the lower jaw

In orthodontic treatment with the completely customized lingual appliance (CCLA) the appliance is manufactured for each patient with individual designed brackets and archwires (Fig. 1).

It was demonstrated that the tooth positions after orthodontic treatment with a CCLA are very close to the planned tooth positions of this set up [1, 2]. A previous study showed that the CCLA is able to provide a very precise torque control especially of the frontal teeth [3]. The precise control of the axis of the front teeth can be especially necessary, if a single tooth needs to be corrected. A specifically single tooth correction is for example needed, if one tooth exhibits a torque problem. Torque describes the tooth axis in the oral-vestibular direction. A torque problem can be caused by late growth of the mandible, occlusal forces or relapse after orthodontic treatment [4–7].

After orthodontic treatment a common method to prevent a relapse in the frontal area is to bond a fixed retainer. This is bonded on the lingual tooth surfaces especially in the lower arch from canine to canine. However, the retainers might have negative effect when they are partially debonded and unnoticed by the patient for a longer time. The teeth rotate around the retention wire either to the buccal or lingual site. There are some studies reporting about undesirable tooth movements due to a broken retainer [8, 9]. These tooth movements can result into a relapse similar to the situation before orthodontic treatment or into a new malocclusion [10, 11].

The orthodontic correction of this torque problem can be a chance to save the tooth and improve the gingival recession. There are some studies reporting about the orthodontic correction of this problem with a vestibular appliance. Different techniques are described in these case reports. Some authors use the technique with segmented archwires, some prefer the technique with continuous archwires [9, 12]. The challenge is to move only the one tooth while not affecting the neighboring teeth, because they are in the correct position. Thus for using a continuous archwire technique a very precise appliance is necessary to control the position of the neighboring teeth. The CCLA is providing a very high precision with a very high torque capacity [13]. So far there is no study published regarding the efficacy of a CCLA for a single tooth torque correction.

Aim of this study was to analyze the efficacy and precision of the CCLA regarding the single tooth torque correction by comparing the plaster casts before and after orthodontic treatment and the set-up models.

The analysis was performed using an optical 3D scanner, digital superposition and measurements of possible gingival recessions on the plaster casts. We also examined external apical root resorptions by orthopantomogram measurements as possible side effects of torque correction and the changings of the periodontal situation.

## Methods

### Patient selection and inclusion criteria

Three patients (females *n* = 2; males *n* = 1), being treated at the Department of Orthodontics of the University of Mainz and private praxis Prof. Dr. Dr. h.c. Wiechmann and partners in Bad Essen (both Germany), were included by the following criteria:

### Inclusion criteria

- single tooth torque problem
- periodontal recession at the lingual or buccal site
- completed treatment with a completely customized lingual appliance
- orthopantomogram and before and after treatment
- plaster casts and pictures before and after treatment
- plaster cast set up

### Treatment procedure

The three patients were treated with a completely customized lingual appliance (WIN, DW Lingual Systems, Bad Essen, Germany). Treatments of all patients were performed with a general archwire sequence of 0.014″ SE-NiTi, 0.016″ X 0.022″ SE-NiTi, 0.016″ X 0.024″ Stainless Steel and 0.018″ X 0.018″ ß-Ti.

### Optical 3D scanner

A 3D Scanner (OrthoXscan, Dentaurum, Germany) was used for the digitalization of the plaster casts. Plaster casts of the situation before treatment, from the set up and from the situation after treatment were fixed on special plates, which are necessary to hold the plaster casts during scan process. These fixators are able to rotate, which allows the three dimensional scanning of the models.

The scanner is composed of a projector and a camera and works with the method of a stripe projection. The projector produces light stripes from different positions, which are registered by the camera. This is necessary to calculate the three dimensional data. All measurements are composed to a complete set of data by a process called “matching” and saved as a Standard Tessellation Language (STL) – file. The. STL-file is a key file for different systems of the Computer Aided Design (CAD)-system.

### Software

The program EasySLIM (3D–Shape, Erlangen, Germany) converted the. STL-files to .pmh-files. In the next step artifacts and unnecessary areas were eliminated by the 3D–Viewer (3D–Shape, Erlangen, Germany).

### Digital superposition

The digital superposition was done with the program Comparison (3D–Shape, Erlangen, Germany). The teeth, which were not included into the appliance and not moved by the orthodontic treatment, functioned as stable structures for the superposition. Everything except of these tooth surfaces was cut before proceeding with the superposition.

**Fig. 2:**
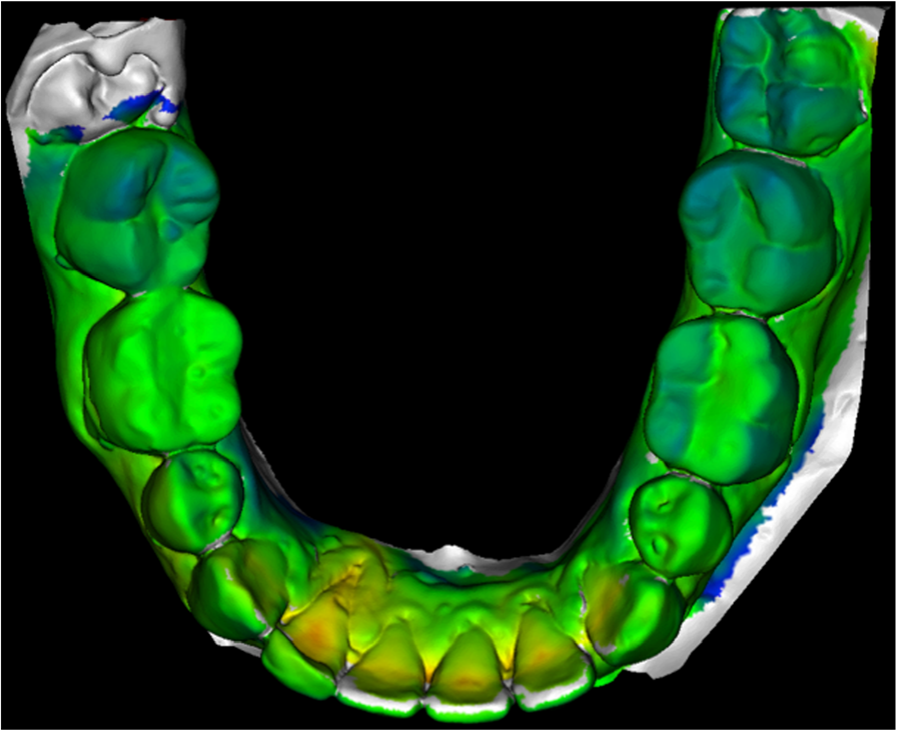
Representation of a superposition of two plaster casts (before and after treatment). Different colors show the amount of position change, which can be measured by the software at any point of interest

The deviations between two scanned plaster casts were shown by colored pictures and metric values of the deviations were calculated by the software. For the optical representation a color scale has been set up. Negative deviations were colored in yellow and red, positive deviations in blue and purple. Area with no or very low deviation were marked green. White areas of the superposition show sections, where the difference between the two scanned models are undetectable for the software (Fig. 2). The Vertex-information-tool gave the information about the value of the deviation. By marking the areas with the mouse the program automatically calculated the value for the deviation. Different points of the tooth arch were then analyzed and the deviations in every axis of the coordinate system were observed. The points of interest were middle of the occlusal surface of the teeth 36 and 46, the top of the canines 33 and 43 and the most apical point of the recession at the affected teeth.

### Measurement of the gingival recession on the plaster cast

Gingival recessions were measured with a caliper gauge on the plaster casts before and after orthodontic treatment. Measurements of the width of the recession were performed at the biggest distance of the recession from the gingival limit mesial to distal. Measurements for the depth were performed from the enamel/cementum border of the crown to the gingival apical limit of recession. All measurements were performed three times.

### Orthopantomogram measurement and treatment time

**Fig. 3:**
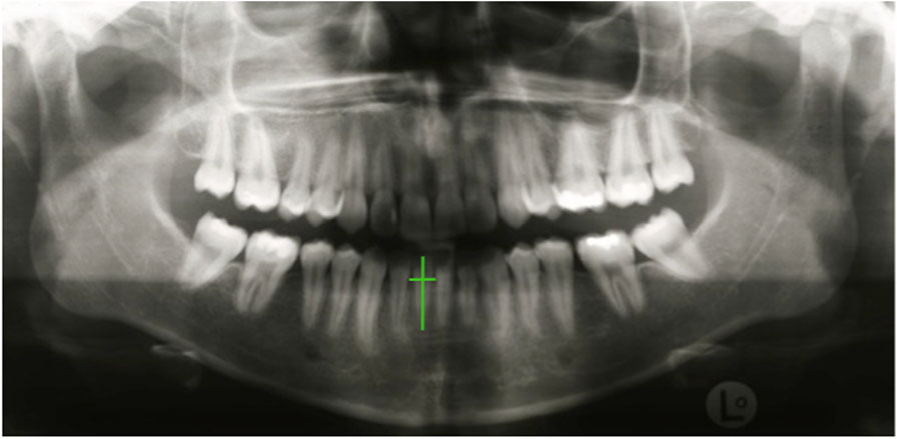
Orthopantomogram of a patient before orthodontic treatment. Lengths of the crown and root of the incisor with the torque discrepancy was measured and compared to the lengths of the crown and the root after treatment

External apical root resorptions (EARR) were defined as any reduction regarding the radiographic root length of the orthodontically moved mandibular incisor. Quantitative measurements of the crown and rot length were taken. Any image distortion between the pre- and post-treatment radiographs was calculated using the crown length measurement [14]. It was decided to express the EARR as relative root resorption seen as the percentage shortening per tooth. Radiographs before and after treatment where performed by the same machine (Fig. 3).

Treatment time were taken out of patients records and clinical pictures were analyzed descriptively.

### Statistical analysis

Results are presented as single values for each patient or as mean values with standard deviations. Differences between the groups are described descriptively.

## Results

### Achieved changes of the root position

**Fig. 4:**
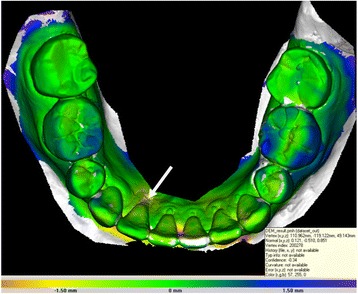
Example of a 3D superimposition of the plaster cast before treatment and after treatment. Different colors show the amount of tooth movement. In this case changes of the most apical point of the lingual recession (white arrow) at tooth 42 were measured

Superposition of the plaster cast before treatment and after treatment showed a mean change of the root of 1.8 mm ± 0.3 mm (patient no. 1: 1.6 mm; patient no. 2: 1.7 mm, patient no. 3: 2.1 mm) into the alveolar bone at the most apical point of the recession (white arrow, Fig. 4).

### Differences between set-up and final model

**Fig. 5:**
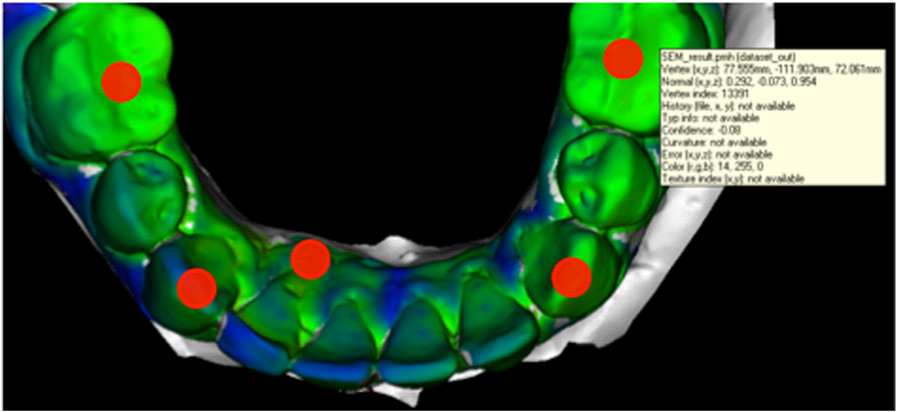
Example of a 3D superimposition of the set up and the final situation after treatment. Different colors show the amount of deviation. Different points of the arch were analyzed and the deviations in every axis of the coordinate system were observed. Target points were on the middle of the occlusal surface of the teeth 36 and 46, the top of the canines 33 and 43 and the most apical point of the recession at the affected teeth

Evaluation of the set-up and final models after treatment showed the deviation between the planned tooth movement and the achieved tooth position (Fig. 5). The highest mean value for the deviation between set up and final model was measured at the occlusal surface of the tooth 36 with 0.8 mm. Nearly all other measurements showed values around 0.5 mm or less (Table 1).

**Table 1:** Mean deviation between the set up and the plaster cast (mm ± standard deviation) of the final situation of every measurement point (occlusal 36 and 46, top of 33 and 43 and at the gingival apical limit of recession) in every axis (X, Y, Z) of the coordinate system

Axis	Occl. 36	Occl. 46	Top 33	Top 43	Apical 42/41
X	0.17 ± 0.14 mm	0.53 ± 0.06 mm	0.43 ± 0.15 mm	0.47 ± 0.06 mm	0.17 ± 0.06 mm
Y	0.4 ± 0.27 mm	0.5 ± 0.3 mm	0.57 ± 0.21 mm	0.77 ± 0.15 mm	0.6 ± 0.2 mm
Z	0.8 ± 0.1 mm	0.53 ± 0.31 mm	0.5 ± 0.3 mm	0.27 ± 0.15 mm	0.56 ± 0.15 mm

### Depths and widths of the recessions

The depths and widths of the periodontal recessions were measured on the plaster casts before and after orthodontic treatment. Mean depth of the periodontal recession before treatment was 8.0 ± 4.6 mm and 3.3 ± 0.5 mm after treatment. Thus the depths were significantly reduced with Δ of 4.7 mm. Mean widths of the recessions was 3.3 ± 0.3 mm before treatment and 2.2 ± 0.3 mm after treatment. Thus Δ of the widths was 1.1 mm. Table 2 shows the values for each patient before and after orthodontic treatment.

**Table 2:** Depths and widths of the recessions (mm) before and after orthodontic treatment (mean ± standard deviation)

Patient	Depth before (mm)	Depth after (mm)	Width before (mm)	Width after (mm)
1	4	2.5	3.5	2.0
2	7.0	4.0	3.5	2.5
3	13.0	3.0	3.0	2.0
mean ± SD	8.0 ± 4.6	3.3 ± 0.5	3.3 ± 0.3	2.2 ± 0.3
Δ change	4.7 mm		1.1 mm	

All values in mm, Δ change means the difference before and after treatment

### Relative external apical root resorption (EARR)

The affected teeth were measured before and after treatment to analyze a possible EARR due to the accomplished torque movement. The mean relative EARR of the three teeth was 2.7 ± 1.5% (patient no. 1: 2.7%; patient no. 2: 4.2%, patient no. 3: 1.2%).

### Treatment time

The treatment time of the patients were analyzed due to the patients` records. Average treatment time was 13.8 ± 4.5 months (patient no. 1: 18.5 months; patient no. 2: 13.5 months, patient no. 3: 9.5 months).

### Clinical situation before and after treatment

**Fig. 6:**
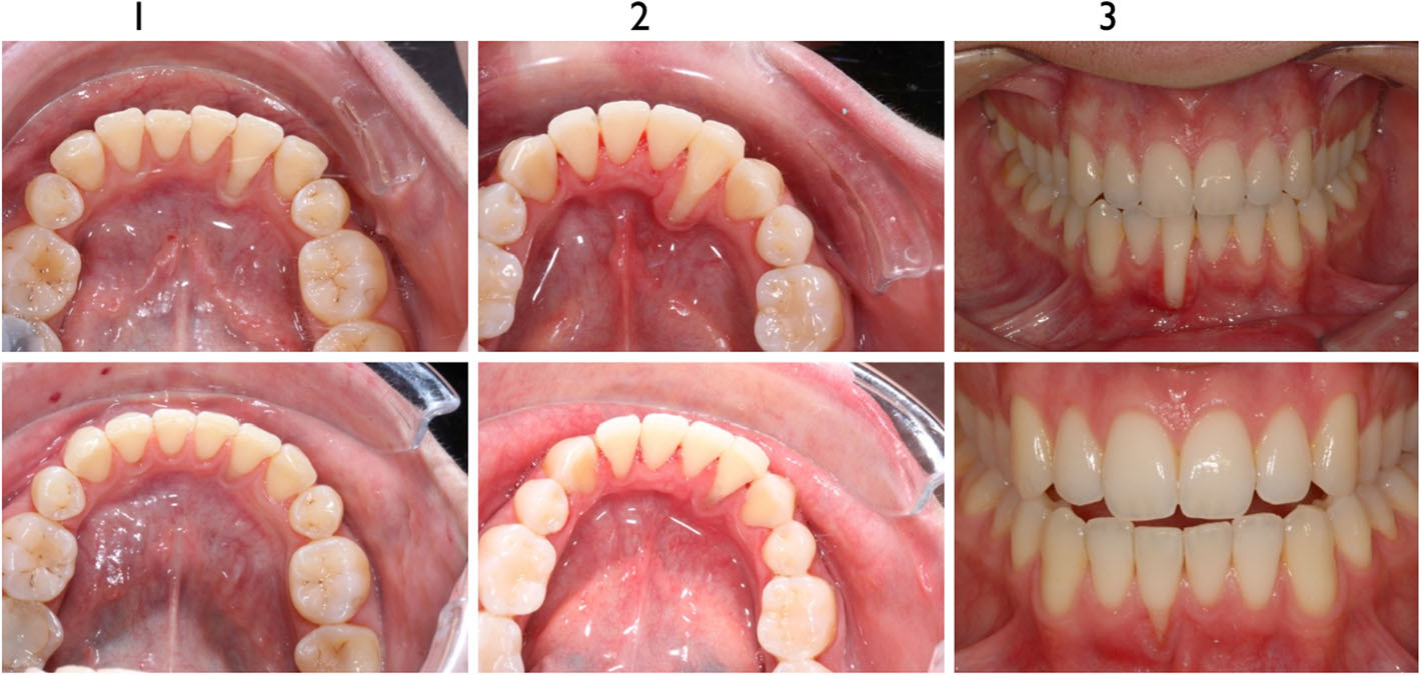
Intraoral clinical pictures of the three patients (I-III) before (upper line) and after orthodontic treatment (lower line)

In the last step we analyzed the clinical situation of the patients by the intraoral pictures. All three patients showed a massive improvement of the periodontal situation after the orthodontic treatment. Torque correction of the affected teeth led to an improvement of the periodontal recession. Two of three patients still had a problem with the hygiene of the gingiva and an inflammation of the gingiva was still observed in these cases (Fig. 6).

## Discussion

The precision of the CCLA has been analyzed in previous studies [1–3]. Pauls et al., 2010 [1], compared set up plaster casts and final model plaster casts of full arch treatments. Consequently all teeth changed their position making the superposition much more complicated. In the present study all patients were treated by a partial appliance. The teeth that were not included in the set up and not integrated into the appliance served as stable structures. Thus, we could use the teeth that were not included into the appliance to superimpose the plaster casts on the surfaces of these teeth. Therefore the gingival parts had to be removed before the process of the superposition.

Pauls et al., 2010 [1], observed a discrepancy between the set up and the plaster cast of the final situation of less than 0.5 mm. In accordance we found out a difference of about 0.5 mm between the two models. This indicates a very high precision and reliability of the methods for the superposition.

The exactness of the 3D scanner is a key factor for the precision of the digitalization of the plaster casts. The orthoX 3D scanner used in the present study provides a precision of less than 20 μm at a scanning time of 45 s. This is the requirement for the quality of the digitalization.

The method to measure the EARR in the orthopantomogram was established by Linge and Linge in 1983 [14] and successfully used in different previous studies [15, 16]. Orthopantomograms are done as a standard procedure before and after orthodontic treatment in Germany. Thus, no additionally x-ray must be done for study reasons. The weakness of the method is the inaccuracy of the orthopantomograms which do not show the apex as sharp as for example a single-tooth radiograph.

In the present study the torque movement of the apex after orthodontic treatment with the CCLA was about 2 mm into the direction of the alveolar bone. The gingival recession improved in all cases with a mean reduction of nearly 5 mm. So far there are only case reports in the present literature describing the periodontal changes after orthodontic single tooth correction. Our results accord with these case reports, which also reported a significant improvement of the periodontal situation due to orthodontic treatment [9, 12]. All these cases showed a massive gingival recession on the tooth with the torque problem before treatment. This underlines the fact that the position of a tooth correlates with its gingival situation. Due to the retrospective study design we were only able to compare the gingival recession by plaster casts and clinical pictures. Further study with a larger amount of patients and clinical measurements of the periodontal situation before and after torque correction are desirable for the future.

Tanaka et al. presented a case report in 2010 and could show a five-year-follow-up of a combined treatment of periodontal surgery and orthodontic treatment for gingival recession in the lower front [17]. The periodontal surgery was performed before orthodontic treatment. Two of the cases presented in our study also underwent periodontal treatment. The periodontal surgery was performed after the orthodontic treatment. The direction of tooth movement might have an impact on the order of the two therapies. Both approaches ended up into very good clinical results. Long time-follow-up-studies are necessary to show a possible advantage of one method.

In our study all cases were under orthodontic retention with a fixed retainer behind the frontal teeth of the mandible. Two of the retainers were partially debonded what the patients did not notice. It is still unclear in the literature where the unexpected tooth movements under partially debonded retainers come from. Either the retainer was bonded with an active component or masticatory forces can be the origin of stress in the retainer wire [10].

Shaughnessy et al. [18] pointed out that fixed retainers are an effective way to prevent a relapse after orthodontic treatment, but require a regular supervision. The wire used as a fixed retainer might have an impact on the risk of unexpected tooth movements, but nearly all wires can create inadvertent tooth movements when they are partially debonded.

To avoid stress between the bonding points of a fixed retainer a novel technique are CAD/CAM-produced lingual retainer. These retainers seem to provide a very accurate fitting to the lingual tooth surface and might prevent unexpected tooth movements caused by residual stress between the bonding points [19]. Future studies are needed to confirm this possible advantage.

We observed a very low external apical root resorption on the teeth after torque correction. Hohmann et al., 2007 [20], described in their finite element study that increased torque resulted in increased high-pressure areas and increased magnitudes of hydrostatic pressure [20]. They concluded, if hydrostatic pressure exceeds typical human capillary blood pressure in the PDL, the risk of root resorption increases. Van Loenen et al., 2007 [21], observed higher root resorptions after the torque movement and described the torque movement as a possible trigger for external apical root resorptions [21]. In our study the teeth, which were torqued, were standing partially out of the bone. Thus, maybe the resistance and hydrostatic pressure during the movement was less and might be the reason for the less observed root resorptions.

## Conclusions

This is the first study showing that the CCLA with its high precision is very effective in correcting single tooth torque problems. Orthodontic torque correction resulted in a significant reduction of gingival recessions and caused only negligible root resorptions.

## Abbrevations

3D: 3 Dimensional
CCLA: Completely customized lingual appliance
EARR: Relative external apical root resorption
SPSS: Statistical Package for the Social Sciences
STL: Standard Tessellation Language

## References

[1] Pauls AH (2010). Therapeutic accuracy of individualized brackets in lingual orthodontics. J Orofac Orthop.

[2] Grauer D, Proffit WR (2011). Accuracy in tooth positioning with a fully customized lingual orthodontic appliance. Am J Orthod Dentofac Orthop.

[3] Lossdorfer S, Schwestka-Polly R, Wiechmann D (2013). Control of lower incisor inclination with a completely customized lingual appliance for dentoalveolar compensation of class III malocclusion. J Orofac Orthop.

[4] Richardson ME, Gormley JS (1998). Lower arch crowding in the third decade. Eur J Orthod.

[5] Rossouw PE, Preston CB, Lombard CJ, Truter JW (1993). A longitudinal evaluation of the anterior border of the dentition. Am J Orthod Dentofac Orthop.

[6] Freitas KM, de Freitas MR, Henriques JF, Pinzan A, Janson G (2004). Postretention relapse of mandibular anterior crowding in patients treated without mandibular premolar extraction. Am J Orthod Dentofac Orthop.

[7] Katsaros C, Livas C, Renkema AM (2007). Unexpected complications of bonded mandibular lingual retainers. Am J Orthod Dentofac Orthop.

[8] Pazera P, Fudalej P, Katsaros C (2012). Severe complication of a bonded mandibular lingual retainer. Am J Orthod Dentofac Orthop.

[9] Farret MM, Farret MM, da Luz VG, Assaf JH, de Lima EM (2015). Orthodontic treatment of a mandibular incisor fenestration resulting from a broken retainer. Am J Orthod Dentofac Orthop.

[10] Sifakakis I, Pandis N, Eliades T, Makou M, Katsaros C, Bourauel C (2011). In-vitro assessment of the forces generated by lingual fixed retainers. Am J Orthod Dentofac Orthop.

[11] Pizarro K, Jones ML (1992). Crown inclination relapse with multiflex retainers. J Clin Orthod.

[12] Machado AW, MacGinnis M, Damis L, Moon W (2014). Spontaneous improvement of gingival recession after correction of tooth positioning. Am J Orthod Dentofac Orthop.

[13] Lossdorfer S, Bieber C, Schwestka-Polly R, Wiechmann D (2014). Analysis of the torque capacity of a completely customized lingual appliance of the next generation. Head Face Med.

[14] Linge BO, Linge L (1983). Apical root resorption in upper anterior teeth. Eur J Orthod.

[15] Jacobs C, Gebhardt PF, Jacobs V, Hechtner M, Meila D, Wehrbein H (2014). Root resorption, treatment time and extraction rate during orthodontic treatment with self-ligating and conventional brackets. Head Face Med.

[16] Sehr K, Bock NC, Serbesis C, Hönemann M, Ruf S (2011). Severe external apical root resorption--local cause or genetic predisposition?. J Orofac Orthop.

[17] Tanaka OM, Avila AL, Silva GM, Añez MC, Taffarel IP (2010). The effects of orthodontic movement on a subepithelial connective tissue graft in the treatment of gingival recession. J Contemp Dent Pract.

[18] Shaughnessy TG, Proffit WR, Samara SA (2016). Inadvertent tooth movement with fixed lingual retainers. Am J Orthod Dentofac Orthop.

[19] Wolf M, Schumacher P, Jäger F, Wego J, Fritz U, Korbmacher-Steiner H, Jäger A, Schauseil M (2015). Novel lingual retainer created using CAD/CAM technology: evaluation of its positioning accuracy. J Orofac Orthop.

[20] Hohmann A, Wolfram U, Geiger M, Boryor A, Sander C, Faltin R, Faltin K, Sander FG (2007). Periodontal ligament hydrostatic pressure with areas of root resorption after application of a continuous torque moment. Angle Orthod.

[21] Van Loenen M, Dermaut LR, Degrieck J, De Pauw GA (2007). Apical root resorption of upper incisors during the torquing stage of the tip-edge technique. Eur J Orthod.

